# Dynamic Acoustic Levitator Based On Subwavelength Aperture Control

**DOI:** 10.1002/advs.202100888

**Published:** 2021-06-09

**Authors:** Xiaolong Lu, Jens Twiefel, Zhichao Ma, Tingting Yu, Jörg Wallaschek, Peer Fischer

**Affiliations:** ^1^ Max Planck Institute for Intelligent Systems Heisenbergstr. 3 Stuttgart 70569 Germany; ^2^ State Key Laboratory of Mechanics and Control of Mechanical Structures Nanjing University of Aeronautics and Astronautics Nanjing Jiangsu 210016 China; ^3^ Institute of Dynamics and Vibration Research Leibniz Universität Hannover An der Universität 1 Garbsen 30823 Germany; ^4^ Institute of Physical Chemistry University of Stuttgart Pfaffenwaldring 55 Stuttgart 70569 Germany

**Keywords:** contact‐free manipulation, dynamic levitation, ferrofluid, subwavelength, ultrasound

## Abstract

Acoustic levitation provides a means to achieve contactless manipulation of fragile materials and biological samples. Most acoustic levitators rely on complex electronic hardware and software to shape the acoustic field and realize their dynamic operation. Here, the authors introduce a dynamic acoustic levitator that is based on mechanically controlling the opening and (partial) closing of subwavelength apertures. This simple approach relies on the use of a single ultrasonic transducer and is shown to permit the facile and reliable manipulation of a variety targets ranging from solid particles, to fluid and ferrofluidic drops. Experimental observations agree well with numerical simulations of the Gor'kov potential. Remarkably, this system even enables the generation of time‐varying potentials and induces oscillatory and rotational motion in the levitated objects via a feedback mechanism between the trapped object and the trapping potential. This is shown to result in long distance translation, in‐situ rotation and self‐modulated oscillation of the trapped particles. In addition, dense ferrofluidic droplets are levitated and transformed inside the levitator. Controlling subwavelength apertures opens the possibility to realize simple powerful levitators that nevertheless allow for the versatile dynamic manipulation of levitated matter.

## Graphical abstract

A dynamic acoustic levitator containing subwavelength apertures permits the generation of complex trapping potentials whilst using only one ultrasonic transducer. Surprisingly, even time‐varying Gor'Kov potentials can be achieved due to the mechanical coupling of the levitated object and a block that covers an aperture. This facile and inexpensive approach opens new capabilities in the field of contactless acoustic manipulation.

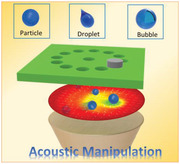

## 1 Introduction

Levitation is an attractive contactless manipulation strategy to handle matter across a wide range of sizes ^[^ ^1^, ^2^, ^3^ ^]^ with diverse applications ranging from biomedicine, analytical chemistry, nanotechnology, and advanced manufacturing.^[^ ^4^, ^5^, ^6^ ^]^ Levitation is particularly promising for fragile materials or bio/chemical samples that are otherwise easily contaminated. Suspending these in mid‐air or inside a liquid and preventing direct contact with container walls prevents their contamination. A number of distinct approaches have been explored to obtain effective levitation.^[^ ^7^ ^]^ Optical tweezers are an invaluable tool in handling colloids, bacteria and cells in solution via noncontact trapping.^[^ ^8^ ^]^ Meanwhile, other platforms utilizing hydrodynamic, plasmonic, magnetic, and electrokinetic forces have been developed, but most of them are constrained by complex instruments or require specific material properties. Acoustic levitation relies on the acoustic radiation force to manipulate particles and is therefore unique in its ability to handle a wide range of materials and to also handle larger objects ranging from 100 nm to cm with acoustic waves from 1 kHz to 500 MHz.^[^ ^9^, ^10^, ^11^, ^12^, ^13^, ^14^, ^15^ ^]^

The conventional levitation system consists of a single‐axis levitator that comprises an ultrasound emitter and an opposing reflector, which establishes a strong standing wave in which objects are trapped at the fixed acoustic pressure nodes.^[^ ^16^, ^17^, ^18^, ^19^, ^20^, ^21^ ^]^ In particular, one floating droplet trapped at a node of a standing ultrasound wave could serve as a containerless reactor for chemical reactions.^[^ ^22^ ^]^ Recent advances include acoustic hologram devices^[^ ^23^, ^24^, ^25^, ^26^ ^]^ and passive modulators developed from acoustic metamaterials^[^ ^27^, ^28^, ^29^, ^30^, ^31^, ^32^ ^]^ that can provide the required phase profiles for arbitrary wave beams to manipulate particles in intricate patterns. However, it is still quite challenging for such systems to dynamically modify target locations on demand. To address this, the dynamic acoustic levitation has recently been demonstrated that is capable of performing particle manipulation and orientation in three dimensions.^[^ ^33^, ^34^ ^]^ A phased array of ultrasonic transducers (PAT) is thus by far the most general way to effectively accomplish real‐time motion control of levitated objects.^[^ ^35^, ^36^, ^37^ ^]^ While the PAT platform shows great flexibility in controlling the trajectory of levitated objects, it is also rather complex and requires the use of several transducers that need to be individually powered and addressed.^[^ ^38^, ^39^ ^]^

Here, we present a manipulation mechanism that can transform the well‐known single‐axis levitator into a dynamic acoustic levitator via the control of subwavelength apertures. Its main advantages are its simplicity and the ability to achieve dynamic levitation with a single, high‐power transducer. Subwavelength apertures are mechanically manipulated to give rise to dynamic trapping fields. The motion of the trapped object inside the levitator can give rise to feedback that causes oscillations and rotations in the trapping potential without external intervention. A number of objects are successfully manipulated inside this setup and indicate its potential for practical applications.

## 2 Results and Discussion

**Figure 1:**
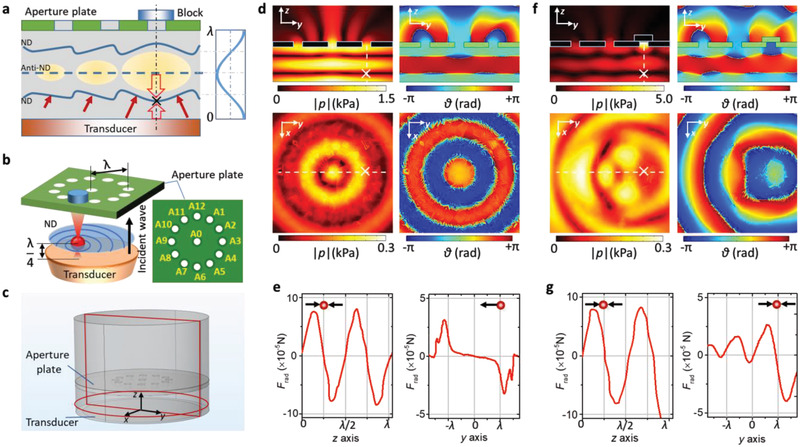
Working mechanism of the dynamic levitator. a) Schematic of working principle. Trapping location is marked by a black cross. The top plate shows two open and one blocked aperture. Nodal planes (ND) and antinode planes (Anti‐ND) of the acoustic standing‐wave are indicated. The wave of the acoustic field is shown for comparison on the right. b) Selected trapping and manipulation of a levitated particle (red) in the nodal plane (ND, light blue) which lies between the transducer (orange) and the aperture plate (green). c) Geometry of the calculated acoustic field planes represented by dark red borders. The *yz*‐plane passes through the center of the setup. The *xy*‐plane is located at a distance of ¼ wavelength from the transducer. d,f) Acoustic pressure and phase distribution, respectively, without and with a blocked aperture A3 (see b). The white cross denotes the corresponding locations in the two planes. The radiation force is calculated along the dashed line in the *yz*‐plane and the *xy*‐plane. e,g) Calculated acoustic radiation force that a 3 mm EPS particle (red) experiences e) with open aperture or g) with blocked aperture A3, respectively. Black arrows indicate the direction of radiation force.

We introduce a structured reflector with subwavelength apertures that affords dynamic manipulation. It overcomes the immobility of particles in a conventional levitator system, where the particles are trapped at the geometrically fixed nodal planes due to a flat reflector. The working principle is shown in **Figure** **1**a. The reflector contains several openings (apertures). It is placed at a distance of one wavelength above the surface of an ultrasound transducer, which acts as the emitter. A standing acoustic wave‐field with two nodal (ND) planes and a single anti‐nodal (Anti‐ND) plane forms between the aperture plate and the transducer. Blocking and (partially) unblocking aperture(s) changes the pressure distribution and introduces asymmetries that can be used for the lateral manipulation of levitated objects. Blocking an aperture deforms the otherwise flat ND plane, and introduces a new stable trapping point below the blocked aperture. Based on this principle, we have constructed one reflector with a circular pattern of 12 apertures (see Figure 1b). The apertures, A1 to A12, are positioned at exactly one wavelength away from the center aperture A0. All apertures have the identical diameter of 1/4 wavelength of the acoustic wave. The bottom ND plane is selected for levitation and the location for the levitated particles is controlled by blocking and unblocking apertures.

Numerical simulations (conducted with COMSOL Multiphysics 5.3) were used to calculate the corresponding acoustic field distributions. Figure 1c shows a sketch of the three‐dimensional simulation model, and the acoustic field is shown for two cross sectional planes (*yz* and *xy*). For details of the numerical model please refer to the supporting information. The transducer is assumed to vibrate with an amplitude of 1 µm and the driving frequency is set to 28.3 kHz, in order to match the experimental conditions. The aperture (open or blocked) is assumed to be perfectly absorbing. The spatial distribution of the acoustic field, including the pressure magnitude and phase angle at different opening conditions, has been calculated and the results are shown in Figure 1d,f. When all apertures are open, as depicted in Figure 1d, a perfectly symmetrical standing‐wave acoustic field is formed in the *yz* plane with the incident wave partially transmitting through the apertures. In contrast, if one aperture (e.g., A3) is blocked, an acoustic pressure gradient is formed underneath the aperture A3.

The acoustic radiation force on a particle inside the setup was calculated using the Gor'kov potential defined as *U*. For small spherical particles in air, it is given by^[^ ^40^, ^41^ ^]^

(1) $$U=\frac{4\pi a^{3}}{3}\left[f_{1}\frac{1}{2}\kappa_{0}\langle p_{\mathrm{in}}^{2}\rangle -f_{2}\frac{3}{4}\rho_{0}\langle v_{\mathrm{in}}^{2}\rangle \right]$$

With the material‐based prefactors *f*_1_ and *f*_2,_ respectively, expressed as

(2) $$f_{1}=1-\kappa_{p}/\kappa_{0}$$

(3) $$f_{2}=\frac{2(\rho_{p}/\rho_{0}-1)}{2\rho_{p}/\rho_{0}+1}$$

Here *p*_in_ and *v*_in_ are, respectively, the acoustic pressure and velocity of the incoming wave, *κ*_p_ and *κ*_0_ are the compressibility of the particles and air, *ρ*_p_ and *ρ*_0_ are the density of the particles and air, *a* is the radius of the particle.

The resultant acoustic radiation force ***F***_rad_ is obtained from the acoustic potential^[^ ^40^, ^41^ ^]^

(4) $$F_{\mathrm{rad}}=-\nabla U$$

The acoustic radiation force on a 3 mm Expanded Polystyrene Sphere (EPS) particle (material properties listed in the Supporting Information), is calculated using Equation (4) for an open aperture A3 (see Figure 1e), and for a blocked aperture (Figure 1g). When the aperture is open, a particle that is located at the position (0, *λ*, *λ*/4) will therefore experience a force that points to the center point (0, 0, *λ*/4) due to the negative radiation force component along the y axis. In contrast, the blocked aperture A3 in Figure 1g results in a lateral force pointing to the position (0, *λ*, *λ*/4). In addition, the Gor'kov potential coupled with the 2D radiation force vectors in the *xy* plane is plotted in Figure S1 of the Supporting Information. These results indicate that objects levitated in acoustic fields can be manipulated and relocated to new positions by controlling the opening of subwavelength apertures. In Figure S1b,d (Supporting Information) the Gor'kov potential as a function of the aperture diameter is shown. It is seen that both the gradient and the size of the trap change with the size of the aperture. Apertures with a diameter corresponding to a ¼ wavelength are able to generate a strong trapping zone with a sharp circular boundary and a deeper trap than for other sizes, which is advantageous for the manipulation of the levitated objects.

**Figure 2:**
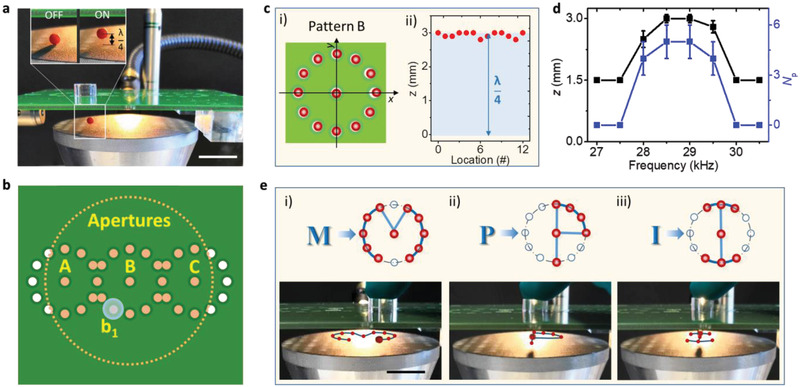
EPS particle levitation and positioning by blocking apertures at different locations. a) Levitation of an EPS particle by switching the applied ultrasound ON/OFF. b) Top view of the experimental setup, including the polymethyl methacrylate (PMMA) block b1 (transparent blue, top layer), the aperture plate (green, middle layer) fabricated with three identical aperture patterns (A, B, and C) and the ultrasound transducer (pink, bottom layer) placed below the aperture plate. The pink dashed circle shows the position for the transducer. c) Manipulation test for trapping (i) and levitating (ii) a single particle by individually blocking apertures at different locations. d) Dependence of levitation height (*z*) and capacity (*N*_p_, number of trapped particles) upon the ultrasound frequency, error bars represent the standard deviation and each group was tested with 5 times. e) Dynamic transport for a levitated particle enabled by moving the block to trace the letters M (i), P (ii), and I (iii). Scale bar: 20 mm.

The experimental setup to demonstrate the dynamic levitation is displayed in **Figure** **2**a. In particular, the solid flat aperture plate (100×100×1.6 mm^3^) is a standard printed circuit board (PCB). The metal‐epoxy‐metal stacked multilayer of the PCB offers good insulation for acoustic waves and is sufficiently stiff to maintain its shape during operation. There is large acoustic impedance mismatch between air (≈4 × 10^2^ kg m^−2^ s^−1^) and PCB (≈6.7 × 10^6^ kg m^−2^ s^−1^), thus we assume perfect reflection at the boundary. A top view of the arrangement is shown in Figure 2b. The acoustic fields generated by the transducer vibration are calibrated when the distance between the aperture plate and transducer is adjusted to *λ*. By measuring the acoustic pressure above the aperture A0 and the vibration amplitude of the transducer for different driving conditions (see Figure S2, Supporting Information), we corrected for a slight discrepancy (< 5%) between the measured and calculated pressure values (with 1 µm vibration amplitude and 28.3 kHz frequency), when the electric power applied to the transducer is around 12 W. The operation time is limited to less than 10 min to avoid heating and to permit open‐loop operation (see the Experimental Section). The inset of Figure 2a illustrates that the levitation for a single EPS particle (diameter: 3 mm; mass: 0.4 mg) can be controlled by switching the ultrasound field ON/OFF. The simultaneous levitation of multiple EPS particles is shown in Figure S3 and Video S1 (Supporting Information).

It is now shown that the apertures give rise to localized trapping in pattern B by individually blocking specific apertures. It can be seen from Figure 2c that the EPS particles localize accurately underneath each blocked aperture. Regardless of different trapping locations, the particle is always levitated at an approximate height of 3 mm (*λ*/4). At resonance, a maximum of 5 EPS particles can be trapped under a single blocked aperture (Figure 2d). The Gor'kov potential shown in Figure S1b (Supporting Information) has a finite region that can tightly trap approximately 5 particles for geometric regions.

Dynamically closing and opening apertures can be used to manipulate the levitated EPS particles within the setup. As shown in Figure 2e (also see Video S2, Supporting Information), we manipulated the apertures of the setup in such a way that the EPS particle was moved along the predefined manipulation paths represented by three letters M, P, and I. The distance between two neighboring apertures is a critical factor that affects the motion of the trapped object (both in continuity and accuracy). We found that a smooth movement with a high motion accuracy is achieved, if two adjacent apertures have a distance of *λ*/2 (6 mm). We found experimentally for the levitated objects of this proposal, that the blocks should be moved at a rate not exceeding 10 mm s^−1^.

**Figure 3:**
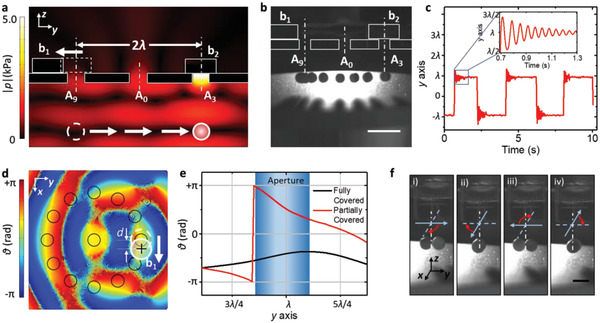
Dynamic response of levitated particles. a) Schematic to induce “jumping” due to locally enhanced acoustic pressure gradients by removing block b1 while keeping b2 at its location. b) Superimposed images taken from Video S3 illustrating the linear motion from the site underneath A9 to A3 via A0. Time lapse: 15 ms. Scale bar: 12 mm. (c) Repeated translation by periodically moving/holding blocks b1 and b2. The inset shows the transient response of overshooting and attenuation when the particle reaches the target location. (d) Schematic for in‐situ high speed rotation, even though the aperture blocks are not moved. A varying phase profile is generated by partially covering an aperture. The trapping center is represented by a black cross mark. (e) Phase angle distribution along the y axis in the vicinity of fully covered and partially covered apertures, respectively. (f) Sequential images captured from Video S4 that show the in‐situ clockwise rotation of levitated particles around the *z* axis. Time lapse: 40 ms. Scale bar: 5 mm.

The apertures of the setup can also be manipulated in such a way that the levitated particles move laterally over larger distances. **Figure** **3**a schematically depicts how levitated objects jump towards new target positions by removing/holding two blocks b1 and b2. It can be seen that block b1 and b2, respectively, cover apertures A9 and A3. A trapped particle below aperture A9 can be released by removing block b1. Due to the planar radiation force shown in Figure 1g the particle jumps along the y axis, until it reaches the target position below the blocked aperture A3. Balanced by the net radiation force due to block b2, the particle will eventually settle at this new equilibrium point. Superimposed images in Figure 3b (sequentially captured from Video S3, Supporting Information) illustrate the fast linear locomotion for an EPS particle. Within a short time of 100 ms, the particle is driven to move linearly from A9 toward A3 at a comparatively high speed of 240 mm s^−1^. The movement can be repeated by periodically moving b1 and b2 (see Figure 3c). The particle first overshoots and then settles within 600 ms (see inset of Figure 3). If several apertures symmetrically located around A3 are also blocked, i.e., A2, A3, and A4, then the trapping regions is larger, as can be seen in the numerical simulation of Figure S4 (Supporting Information). Therefore, the target region is less well defined. If two separated particles are to be manipulated simultaneously, then our experimental results indicate that these should be separated by at least one wavelength to ensure separated trapping zones.

While the above movements almost exactly follow the blocking and unblocking of the apertures, it was surprising to observe sustained dynamic motion without actively changing the state of the apertures. For instance, the rotation and orbiting of levitated objects was observed when apertures were covered only partially. As the simulation results shown in Figure 3d illustrate, a partially covered aperture (block b1 moved to a distance *d* of ≈1/8 wavelength along the *x* axis), results in a phase gradient (Figure 3e).

A particle that is trapped below a partially covered aperture experiences an in‐plane torque and rotates around the trapping center.^[^ ^37^ ^]^ Compared to the phase distribution generated by a fully blocked aperture shown in Figure 3e, there is a comparatively high phase gradient for the partially covered aperture. Such a phase gradient in turn creates a time‐changing local tangential radiation force below the aperture on half of the particle. The partial blocking also changes the geometrical center of the particle dimer, which will move closer to the trapping center and in turn is not aligned with the center of the covered aperture. Hence, the unsymmetrical tangential radiation force will induce a net in‐plane torque, which results in an angular momentum transfer and the rotation of the particle. In Figure 3f, sequential images taken from Video S4 (Supporting Information) display one cycle of an in situ rotation by two particles. The two particles forming a dimer rotate stably (in a clockwise direction) at 350 ±10 rpm. The fields here can give rise to a rich phenomenology due to a locally changing Gor'kov potential gradient. As a consequence, the aperture blocking method with one single‐axis transducer is promising to develop robust and easy‐to‐use dynamic manipulation platforms, as the electronics is simple and compatible with ultrasonic transducers and as the aperture masks can be designed with increasing complexity.^[^ ^17^, ^25^, ^42^ ^]^

**Figure 4:**
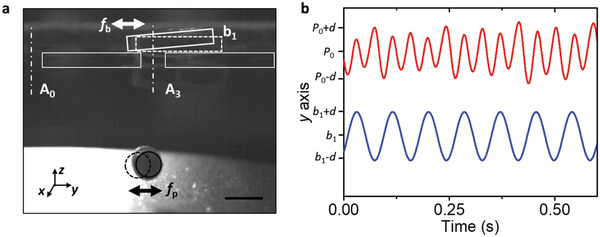
Self‐excited low frequency oscillation due to coupling of the blocking and trapping potentials. (a) Captured image from Video S5 showing the oscillation of a levitated particle at *f*_p_ frequency triggered by the self‐excited motion of the block b1 oscillating at a frequency *f*_b_. The particle is trapped below the covered aperture A3. Scale bar: 5 mm. (b) Temporal oscillation response (amplitude *d*: ≈ 1.5 mm) showing the particle motion (upper red curve) and the motion of the block (lower blue curve) at different frequencies (*f*_p_: 24 Hz, *f*_b_:12 Hz) around their initial positions (*p*_0_ and *b*_0_), respectively.

Interestingly, a self‐modulating potential was realized when the aperture was covered by a light block (≈210 mg as opposed to a heavier block of ≈360 mg which does not move). An explanation for this phenomenon is that the levitated particle changes the acoustic field, which in turn affects the acoustic radiation force on the block which is used to close the aperture. In our experiment, the light block responded to the acoustic field, which in turn coupled back to the trapped particle. The spring/time constants of both vibrations are generally different, such that it is possible to realize a fully dynamic trapping scenario. The low frequency vibration of the block triggers the in‐plane oscillation of the levitated particle, as is illustrated in **Figure** **4**a. If the block used to close the aperture is not pressed against the reflector plate by an external force, but is supported only by its own weight, the acoustic forces may be large enough to cause it to lift off temporarily. This lift partially opens the aperture, which in turn reduces the acoustic pressure, and the block settles again because its gravity dominates over the acoustic radiation force. The dynamic interplay between radiation force and gravity gives rise to an oscillating motion of the block. As shown in Figure 4b, the oscillating amplitude and frequency are 1.5 mm and 12 Hz, respectively. Coupled to the vibration of the light block, the particle experiences varying trapping forces and accordingly oscillates at a frequency of 24 Hz. The oscillation of the levitated particle can be affected by the strength of the ultrasound field as it determines the in‐plane stiffness of the trap.

**Figure 5:**
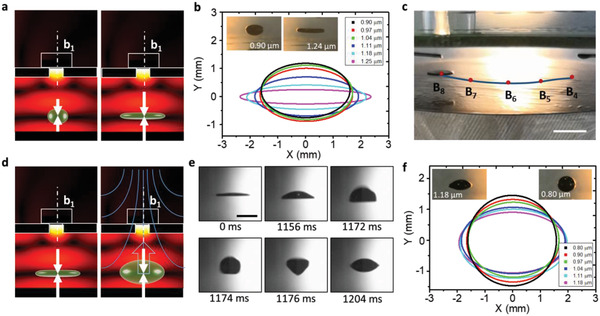
Dynamic deformation of ferrofluidic droplets in acoustic fields. (a) Schematic representation for deforming one levitated droplet (dark green) as a function of increasing radiation force (white arrows). (b) Geometrical variation of the levitated ferrofluidic droplet in response to different transducer vibration amplitudes. (c) Dynamic transport for a compressed thin film of ferrofluid by moving the block covering the aperture. Scale bar: 7 mm. (d) Schematic showing the generation of levitated bubbles from a compressed ferrofluidic thin disk with the combination of acoustic and magnetic fields (blue). (e) Sequential images taken from Video S8 illustrating the bubble formation process, including buckling (0‐1156 milliseconds), expansion with rim closure (1172‐1176 milliseconds) and stabilization (after 1204 milliseconds). Scale bar: 5 mm. (f) Geometrical variation of the levitated ferrofluidic bubble as a function of the transducer's vibration amplitude.

Finally, we show that the levitator can also be used to levitate dense ferrofluidic droplets, composed of magnetic nanoparticles. Ferrofluidic drops have become an important tool to investigate droplet dynamics under the influence of an external force.^[^ ^43^, ^44^ ^]^ To date most studies involving ferrofluids, including promising applications in active matter, soft robotics and other programmable liquid systems, have been limited to those where the drop is in contact with a surface.^[^ ^45^, ^46^, ^47^, ^48^, ^49^ ^]^ Acoustic levitation for diverse droplets has been shown,^[^ ^5^ ^]^ and we here explore the capability to levitate ferrofluidic droplets in our manipulation platform. As **Figure** **5**a illustrates, a ferrofluidic droplet can be trapped by the locally intensified radiation force below the aperture covered by block b1. When the radiation force is increased the shape of the drop changes such that it is compressed into a thin disk. By tuning the applied ultrasound pressure, the shape of a ferrofluidic droplet with a net volume of about 10 µL has been systematically varied, as can be seen in Figure 5b. When the vibration amplitude increases from 0.9 to 1.25 µm the cross‐sectional profile of the drop changes from a 2 mm elliptical sphere to a 0.5 mm thin disk. Like other acoustically levitated droplets, the ferrofluidic droplet also changes its shape at high vibration amplitudes (over 1.25 µm).^[^ ^50^ ^]^ With the same strategy for manipulating particles mentioned above, the dynamic translation of the ferrofluidic droplet was experimentally shown by moving the block covering an aperture, as shown in Figure 5c; and Video S6 (Supporting Information).

Interestingly, the setup can also be used to induce a bubble in the trapped and flattened ferrofluidic disk. Figure 5d shows that the compressed thin film, which is levitated by the acoustic field, can buckle when a vertical magnetic field is applied. The buckled liquid film forms a cavity that resonates and eventually encloses a bubble.^[^ ^50^ ^]^ A magnetic field of 6 mT was applied to deform the magnetic droplet, as recorded in Figure 5e. The dynamic transformation from a droplet to a bubble is shown in Video S7 (Supporting Information). The ensuing process via thin film buckling, cavity expansion, and bubble formation was observed in a very short time (<50 ms). Once the bubble forms in the acousto‐magnetic field, it is stable for more than 10 min. Again, it is also possible to change the shape of the bubble, as seen in Figure 5f. Specifically, a perfectly spherical magnetic bubble could be produced and stabilized when the vibration amplitude reached 0.8 µm. This opens the possibility to compensate effects due to gravity in levitated ferrofluidic drops and magnetic bubbles. More detailed bubble shapes at different vibration amplitudes of the transducer are shown in the supporting Figure S5 (Supporting Information). Consequently, our platform is capable of generating levitated magnetic bubbles transformed from spherical ferrofluidic droplets. The levitator platform can therefore be used to study bubble dynamics, which is of interest for fundamental studies,^[^ ^51^ ^]^ as well as in the formation of core–shell materials^[^ ^52^ ^]^ and in the field of optical engineering.^[^ ^53^ ^]^

## 3 Conclusion

In summary, we have demonstrated an acoustic levitator that contains subwavelength apertures in its reflector to permit the dynamic mechanical manipulation of levitated objects. The proposed strategy enables the conventional single‐axis acoustic levitator system to achieve on‐demand contactless transport in a facile manner, instead of using multiple emitters spatiotemporally controlled by complex electric systems.^[^ ^33^, ^54^, ^55^ ^]^ While the presented system is not as general as a PAT‐system, it has the distinct advantage that it can be operated with only one single, ultrasonic transducer. The (partial) blocking of subwavelength apertures can give rise to a time‐varying Gor'kov potential that can be used to realize long distance translation, in‐situ rotation and self‐modulated oscillation without the need for any time‐varying input fields. The proposed subwavelength aperture control thereby provides compelling alternatives for conducting dynamic levitations of different targets (solid particles, droplets, and bubbles) in a facile, inexpensive and easy manner. A potential limitation compared with phased‐arrays of transducers is that the mechanical opening and closing of the apertures in this work is limited to speeds of up to 10 mm s^−1^. In addition, a limitation is that the aperture patterns have to be fabricated ahead of time. We expect that the demonstrated levitation technology holds great promise for contactless manipulation of biological materials, crystals and advanced manufacturing applications, especially in conjunction with high‐power transducers.

## 4 Experimental Section

### Experimental Setup

The experimental system is composed of one solid flat aperture plate, one ultrasound transducer, one pillar‐shaped block, one manual stage, and one illuminator. The Langevin ultrasound transducer (HNC‐4SH‐4528N, Hainertec Co., Ltd, Suzhou, China) was clamped by a customized holder and fixed to the top surface of an optical table. The flat aperture plate was designed using the EAGLE software (AUTODESK, San Rafael, CA) and fabricated with standard printed circuit board technology (AISLER B.V., Limburg, Netherlands). It was fixed to a XZ axis manual positioning stage (LM‐112S, CHUO Precision Industry, Japan). By adjusting the manual stage, the distance between the aperture plate and the transducer could be precisely changed. One fiber illuminator (KL1500 LCD, Leica microsystem GmbH, Wetzlar, Germany) was placed behind the aperture plate to illuminate the setup. The block used to block selected apertures, was cut from a 5 mm polymethyl methacrylate (PMMA) board by a laser cutter (Zing, Epilog Laser, Netherlands). One digital signal generator (JDS‐2900, JUNTEK, Hangzhou, China) was used to create sinusoidal drive signals with a tunable frequency, which was fed to a power amplifier (AG Series, T&C Power conversion, TX) and then applied to the ultrasound transducer.

### Preparation of the Object Samples

Expanded Polystyrene Spheres (SMOOTHY, Germany) with the diameter of 3 mm were used as supplied. Multiple color dyes were surface‐painted on different EPS spheres in order to facilitate their observation and identification. Before the experiments, the size and weight of each particle was checked to ensure the particles had consistent properties. The ferrofluid sample (M‐FER‐10, Supermagnet, Germany) with nano ferromagnetic particles dispersed in hydrocarbonic oil was used for demonstrating the dense liquid manipulation and transformation. The viscosity and density for the ferrofluid sample are quoted as 80 mPa s and 1040 kg m^−3^, respectively.

### Calibration of the Acoustic Field

The measurement system for calibrating the acoustic field is shown in Figure S2a (Supporting Information). The vibration characteristics of the ultrasound transducer were measured by a fiber vibrometer (OFV‐552 utilizing a decoder VD‐09, Polytec GmbH, Waldbronn, Germany). Driving conditions were monitored by an oscilloscope (DSO‐X 2004a, Agilent, CA), working with the voltage probe and current probe (P6021, Tektronix, OR). The acoustic pressure was measured by an optical microphone (Eta250 Ulta, XARION Laser Acoustics GmbH, Austria). During the calibration, the same driving conditions including the voltage and frequency were used for measuring both vibration amplitude and the resultant acoustic pressure. Regarding the limited space between the aperture plate and transducer, the sensor head of the microphone was fixed above the opening aperture A0 with the height of one wavelength (≈12 mm). The laser beam sent from the vibrometer passes through another aperture A3 to measure the vibration of the transducer. To maintain the same resonance operation for the transducer, the applied frequency was slightly tuned to obtain the maximum current value at different driving voltages (see Figure S2b, Supporting Information). The dependence of pressure and vibration amplitude upon the current were tested as shown in Figure S2b,c (Supporting Information). Therefore, the calibrated results between the acoustic pressure and the vibration amplitude were obtained as shown in Figure S2e (Supporting Information). The deviations from linearity around a driving voltage of 25 V is ascribed to intrinsic nonlinearities of the ultrasonic ultrasonic transducer. The current was monitored to ensure that the transducer produces a constant vibration amplitude.

### Imaging and Manipulation Experiments

The ferrofluid manipulation was conducted with a 10 µL solution droplet containing nano ferromagnetic particles that were introduced into the levitation space via a syringe pump (Model 22, Harvard Apparatus, MA). For triggering the transformation of the ferrofluidic droplet, one permanent magnet was assembled to the top of the dynamic levitator system. The magnet field strength in the levitation space was measured by a teslameter (Model 3MH3, Senis AG, Switzerland). To record the fast movement or the deformation for the levitated particles, a high speed camera (S‐motion, AOS Technologies AG, Switzerland) was utilized with a frame rate of 500 fps. To obtain the movement trajectories, captured videos were processed in Image J software. Image reconstruction from the obtained videos was processed using Origin (OriginLab, Northampton, MA).

## Conflict of Interest

The authors declare no conflict of interest.

## Supporting information

Supporting Information

Supplemental Video 1

Supplemental Video 2

Supplemental Video 3

Supplemental Video 4

Supplemental Video 5

Supplemental Video 6

Supplemental Video 7

Supplemental Video 8

## Data Availability

Research data are not shared.
