# Supplementary material for: Dynamic Acoustic Levitator Based On Subwavelength Aperture Control
Source: Adv Sci (Weinh). 2021 Jun 9;8(15):2100888. doi: 10.1002/advs.202100888 (PMC8336493; doi:10.1002/advs.202100888)
Supplement: Supplementary file 1 — Supporting Information [file ADVS-8-2100888-s009.pdf]

WILEY-VCH

© 2021 Wiley-VCH GmbH

ADVANCED SCIENCE
Open Access

Supporting Information

for *Adv. Sci.,* DOI: 10.1002/advs.202100888

Dynamic acoustic levitator based on subwavelength aperture control

Xiaolong Lu, Jens Twiefel, Zhichao Ma, Tingting Yu, Jörg Wallaschek, and Peer Fischer**

Supporting Information

Dynamic acoustic levitator based on subwavelength aperture control

Xiaolong Lu, Jens Twiefel, Zhichao Ma, Tingting Yu, Jörg Wallaschek, Peer Fischer**

Table S1. Material properties for numerical calculation

**Parameters**	**Air host medium**	**EPS particle**
**Density** ρ **(kg/m^3)**	1.18	29
**Speed of sound** c **(m/s)**	346	900
**Compressibility** κ **(MPa^{-1})**	7.08	425
**Radius** a **(mm)**	---	1.5

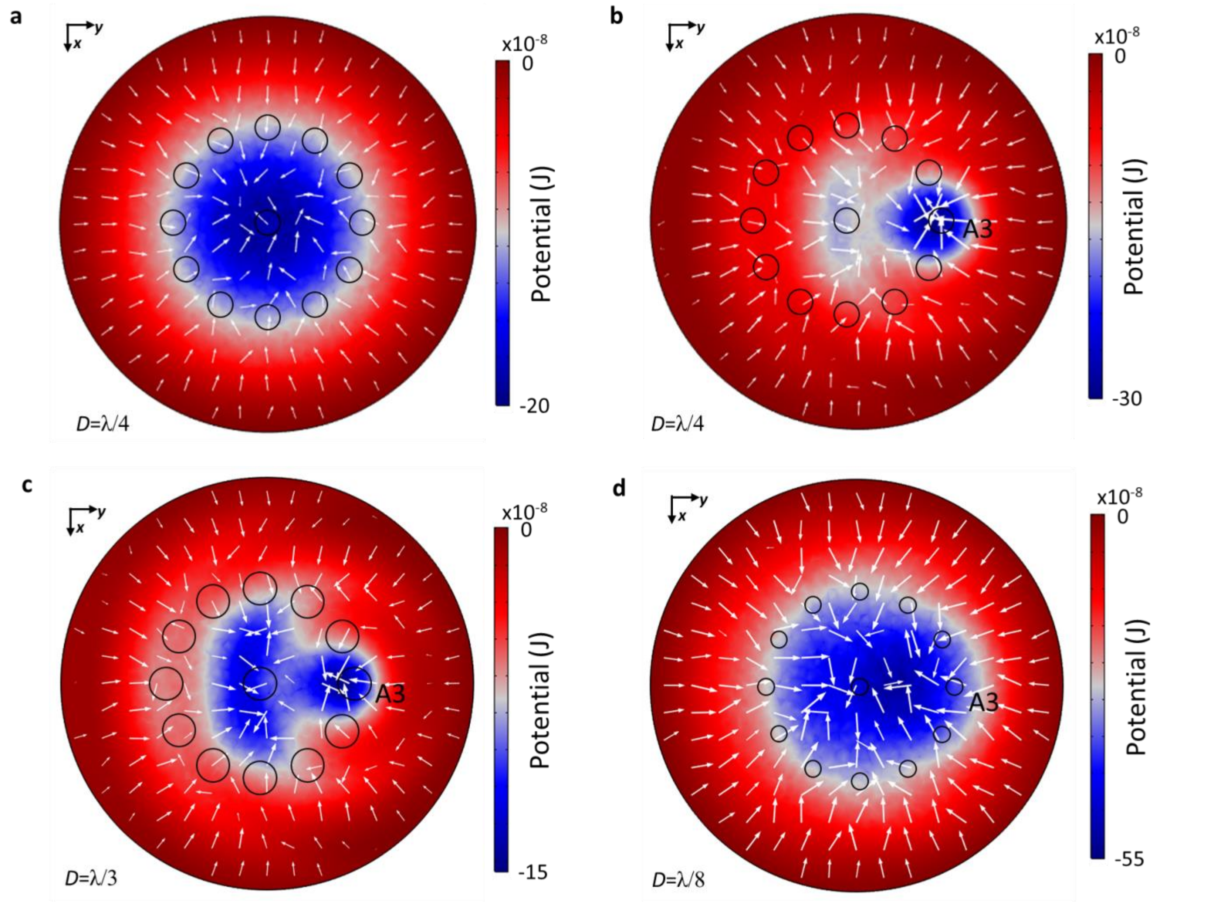

Figure S1 Gor'kov potential distribution in xy plane for a 3 mm EPS particle, with(a) All apertures are kept open, and (b~d) aperture A3 is blocked while the other apertures are kept open with different aperture diameters. Parameter *D* denotes the aperture diameter. The directions for acoustic radiation force is represented by white arrows.

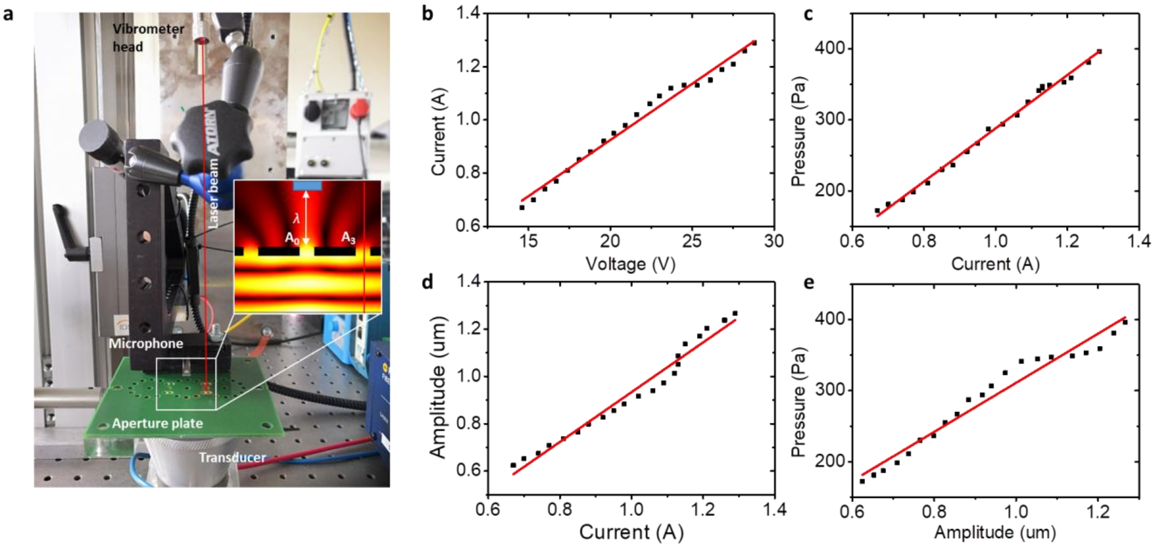

Figure S2 Calibration of the acoustic field at different driving conditions. (a) Experimental setup for the calibration, including ultrasound vibration measured by a fiber vibrometer, acoustic pressure measured by an optical microphone, and the driving signals for the transducer measured by voltage and current probes. The inset displays the optic microphone located on top of the central aperture A0 at a height of one wavelength and the laser beam from the vibrometer head passes through the aperture A3 to measure the vibration from the transducer. (b) Dependence of the driving current upon the driving voltage at resonance. (c) Dependence of the vibration amplitude upon the driving current. (d) Dependence of the acoustic pressure upon the driving current. (e) Dependence of the acoustic pressure upon the vibration amplitude. From figures (b) to (e), black rectangles show the measured results and solid red curves show fits to the data.

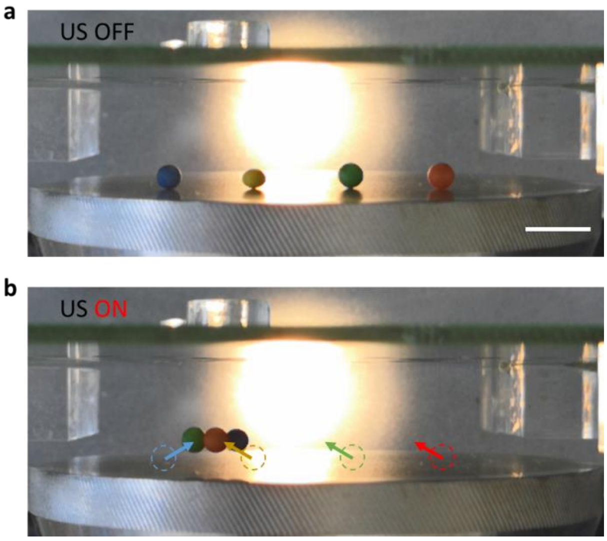

Figure S3 Levitation of several EPS particles. (a) Initial positions for four separated EPS particles when the ultrasound is OFF. (b) Stabilized levitation for the assembly of four EPS particles trapped below the blocked aperture when the ultrasound is ON. Scale bar: 10 mm.

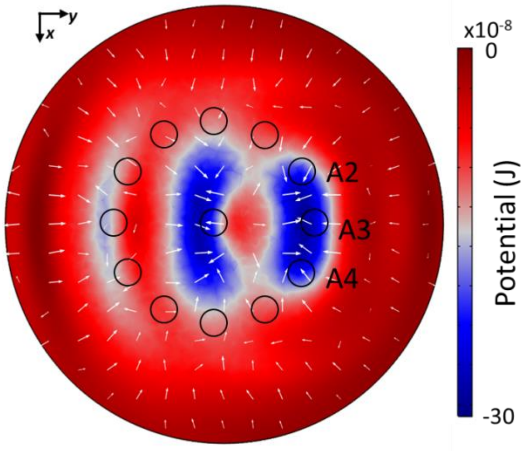

Figure S4 Gor'kov potential distribution in xy plane for three blocked apertures: A2, A3 and A4

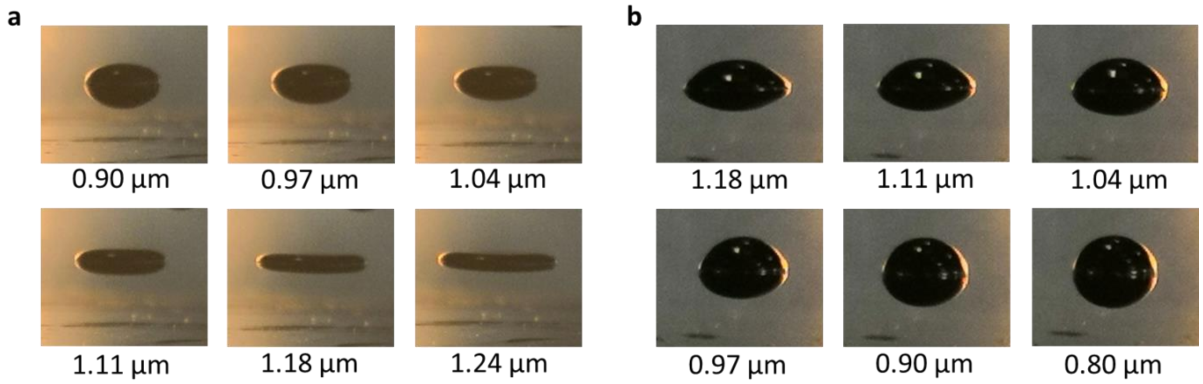

Figure S5 Schematic of ferrofluidic droplets at different vibration amplitudes. (a) Shape of droplet changes from ellipsoidal to a thin disk as the amplitude is increased. (b) Shape of bubble changes from a prolate to a rounded spheroid.

Experimental videos

Video S1. EPS particles levitation via the blocking of apertures.
Video S2. Dynamic transport for a levitated particle.
Video S3. Fast linear locomotion with local acoustic gradients.
Video S4. Phase shifts enable high speed rotations.
Video S5. Autonomous low frequency oscillation.
Video S6. Dynamic transport for a compressed thin ferrofluid droplet.
Video S7. Real-time transformation for a droplet to bubble.
Video S8. Slow motion for bubble generation under acoustic and magnetic fields.
